# Probiotics and the Microbiota-Gut-Brain Axis: Focus on Psychiatry

**DOI:** 10.1007/s13668-020-00313-5

**Published:** 2020-05-13

**Authors:** Sabrina Mörkl, Mary I Butler, Anna Holl, John F Cryan, Timothy G Dinan

**Affiliations:** 1 APC Microbiome Ireland, University College Cork, Cork, Ireland; 2 Department of Psychiatry and Psychotherapeutic Medicine, Medical University of Graz, Graz, Austria; 3 Department of Psychiatry and Neurobehavioural Science, University College Cork, Cork, Ireland; 4 Department of Anatomy and Neuroscience, University College Cork, Cork, Ireland

**Keywords:** Probiotics, Microbiota-gut-brain axis, Gut microbiota, Vagal nerve, Psychiatry, Depression, Schizophrenia, Anxiety

## Abstract

**Purpose of Review:**

Probiotics are living bacteria, which when ingested in adequate amounts, confer health benefits. Gut microbes are suggested to play a role in many psychiatric disorders and could be a potential therapeutic target. Between the gut and the brain, there is a bi-directional communication pathway called the microbiota-gut-brain axis. The purpose of this review is to examine data from recent interventional studies focusing on probiotics and the gut-brain axis for the treatment of depression, anxiety and schizophrenia.

**Recent Findings:**

Probiotics are likely to improve depression but not schizophrenia. Regarding anxiety, there is only one trial which showed an effect of a multispecies probiotic. However, determinants like the duration of treatment, dosage and interactions have not been thoroughly investigated and deserve more scientific attention.

**Summary:**

Microbiome-based therapies such as probiotics could be cautiously recommended for depression to enhance beneficial bacteria in the gut and to improve mood through the gut-brain axis.

## Introduction

The gut microbiota is a complex assembly of bacteria, viruses, protozoa, archaea and fungi which inhabit the human gastrointestinal tract (GIT). The number of bacteria in the body slightly exceeds the number of human body cells [1], and not surprisingly, bacteria are essential for a range of physiological processes. Interestingly, cellular organelles such as mitochondria, the adenosine-tri-phosphate (ATP)-generating power plants of the body, are also of bacterial origin and appear to be related to *Proteobacteria* [2], underlining the central role of bacteria for life, health and disease. The predominant phylotypes in the gut are *Firmicutes* and *Bacteroidetes*, but there is a high, finger-print-like individuality of microbial communities, and the terms of a ‘healthy gut microbiome’ and ‘dysbiosis’ still remain controversial [3].

There is a complex communication system between the GIT, the micro-organisms which inhabit it and the peripheral and central nervous systems (CNS). This is termed the microbiota-gut-brain axis (MGBA) and constantly transmits and interprets information from the periphery to the brain and back. The exact mechanisms of this communication are still under investigation and involve neural (vagus nerve and enteric nervous system), endocrine (cortisol and hypothalamic-pituitary-adrenal (HPA) axis) and immune (cytokine) pathways. It is noteworthy that these pathways are also often found to be altered in the context of psychiatric disorders.

The gut microbiota is a modifiable target with the potential for epigenetic modification [4•] and could therefore be used to treat and ameliorate symptoms of psychiatric disorders. The MGBA can be modified with certain prebiotics (dietary modification/diets rich in non-digestible fibre), probiotics (living bacteria), antibiotics, synbiotics (combinations of pre- and probiotics), postbiotics (bacterial fermentation products such as short chain fatty acids (SCFAs)) and faecal microbiota transplantation (FMT) [5]. All these approaches could be regarded as potential psychobiotics, as they are suggested to improve mental health through their microbiota-modifying properties [6, 7].

Probiotics are live organisms, that when administered in adequate amounts, offer health benefits to the host [8]. The treatment of depression and anxiety with probiotics was first suggested in 1910 [9] and then revisited in 2005 [10]. To date, only a limited number of clinical studies have tested the effects of probiotics on the MGBA and their possible efficacy in the treatment of psychiatric disorders. The purpose of this review is to examine the recent literature on the effects of probiotics on the MGBA and to review data from recently published prospective clinical trials which studied probiotics as a treatment for depression, anxiety and schizophrenia.

## Search Strategy and Selection Criteria

We searched PubMed for original research articles, systematic reviews and meta-analysis conducted over the last 5 years (January 2014–December 2019). The following search terms were used: ‘probiotics’, ‘psychobiotics’, ‘gut-brain axis’, ‘microbiota-gut-brain axis’ and combinations with ‘depression’, ‘anxiety’, ‘social anxiety disorder’, ‘generalized anxiety disorder’, ‘schizophrenia’, ‘inflammation’ and ‘vagus nerve’. Reference lists of relevant articles were also reviewed to find additional literature.

Human studies were included if they were clinical, randomised controlled trials (RCTs). The study population in these papers must have been clinically diagnosed with either depression, an anxiety disorder or schizophrenia. Relevant questionnaires must have been used to quantify psychiatric symptoms (such as the Beck Depression Inventory (BDI) for depression severity). An intervention of probiotics must have been studied. The following exclusion criteria were relevant: case reports with *n* = 1 or a low *n* number have been excluded; studies investigating subjects with no clinically diagnosed mental health condition or no reported intervention with probiotics. For the creation of Table 2, the population, interventions, comparisons, outcomes and study design (PICOS) criteria were used to summarise the research.

## Results

### The Microbiota-Gut-Brain Axis and Its Components

**Fig. 1:**
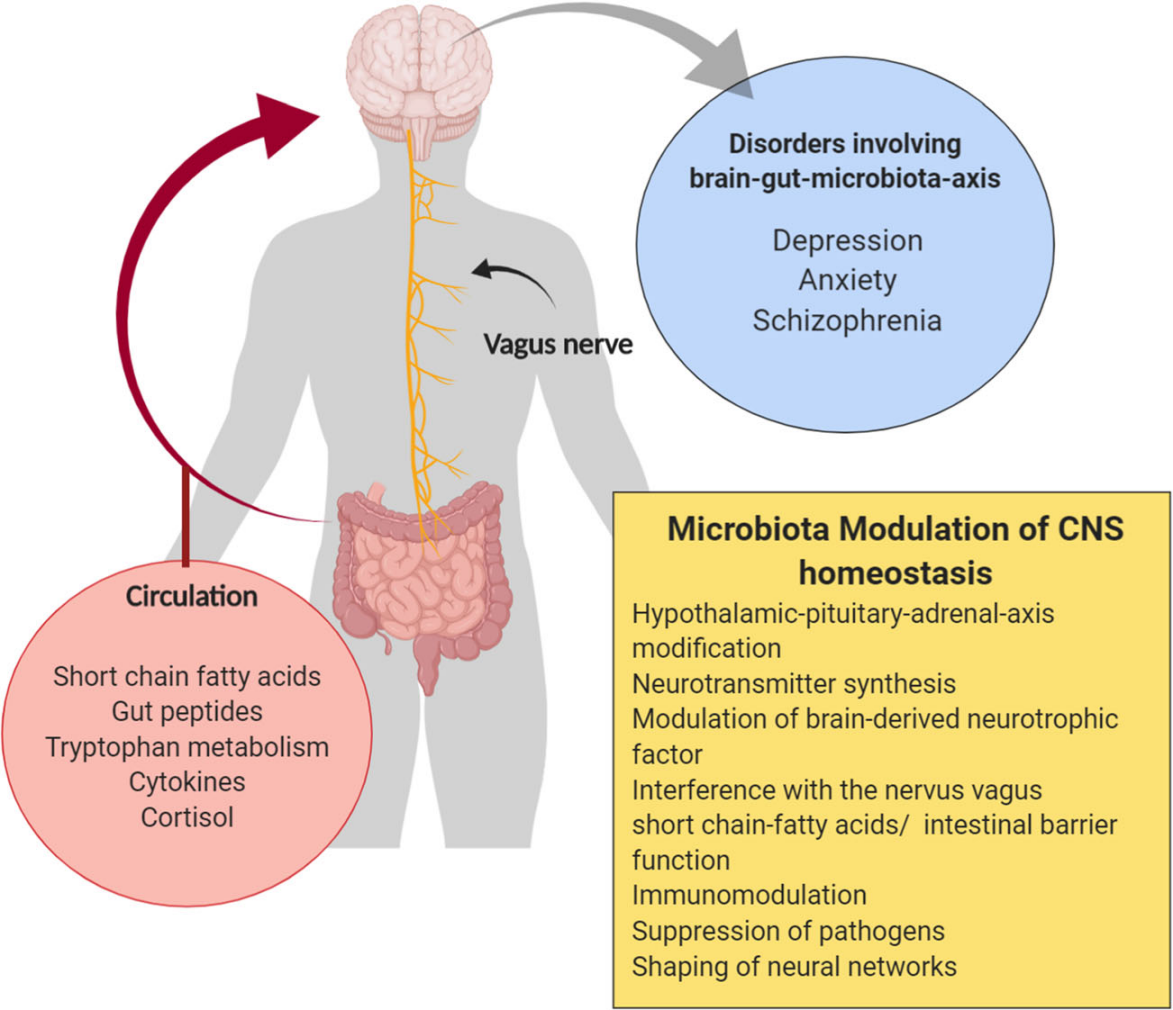
Microbiota modulation of the central nervous system (CNS). This figure was created with BioRender.com

Gut microbes constantly interact with the brain through a range of pathways, including immune regulation, metabolism of neurotransmitters, SCFAs and vagal afferents [21, 22] (see Fig. 1). Further, the gut microbiota determines stress responsivity by influencing the hypothalamic-pituitary-adrenal axis (HPA axis) [23] and stress cortisol responses can be altered by several probiotics [24, 25]. Elevated stress levels are intertwined with anxiety and depression. The rates of depression and anxiety are disproportionally high in patients with functional gut disorders. Mikocka-Walus et al. found that—by including studies examining either symptoms with validated screening scales (i.e. the Hospital Anxiety and depression scale) or the structured clinical interview for DSM—the pooled mean proportion for anxiety in inflammatory bowel diseases versus healthy controls was 19.1 versus 9.6%. For depression, it was 21.2 versus 13.4% [26]. Table 1 lists possible mechanisms of psychobiotics on the gut-brain axis.

**Table 1:** Mechanisms of psychobiotic action

Mechanisms of psychobiotic action	
Hypothalamic-pituitary-adrenal axis (HPA) modification [27]	
Neurotransmitter synthesis (such as gamma aminobutyric acid, serotonin, dopamine, noradrenaline, melatonin, histamine and acetylcholine) [28–30]	
Modulation of brain-derived neurotrophic factor (BDNF) [31]	
Modulation of oxytocin [32]	
Interaction with the 10th cranial nerve (nervus vagus) [33]	
Postbiotics (such as short chain fatty acids) [34, 35]	
Preservation/improvement of the intestinal barrier function [36]	
Training of the immune system, immunomodulation [37]	
Suppression of pathogens [38]	
Shaping of neural networks [39]	

### Psychobiotics

Psychobiotics was initially referred to probiotics causing alterations of mood, anxiety and cognitive function [6]. The term ‘psychobiotics’ now includes all microbiota-targeted interventions such as probiotics and prebiotics that influence bacteria-brain relationships [7•].

Probiotics, living bacteria with health-improving properties are dosed in ‘colony forming units’ (CFU) [8, 40]. In most studies, probiotics such as *Lactobacillus* and *Bifidobacteria* species [41•] are administered but yeast strains (such as *Saccharomyces boulardii*) are also used [42]. Probiotics are thought to contribute to a balanced gut environment by suppressing pathogens and interacting with host microbiota. Some bacterial species are not inherently pathogenic as they are found in small abundances in healthy hosts; however, if they become a dominant species in the gut environment, this leads to a disease. Therefore, a diverse environment is of importance and probiotics are thought to contribute to this diversity. Further, they train the immune system and have effects on metabolism and hormone function [43, 44].

One of the major determinants of the gut microbiota composition is prebiotics and diet. Animal- and plant-based diets cause dramatic shifts of the gut microbiota within days [45, 46]. Certain dietary styles, such as the Mediterranean diet, are rich in plant-based foods and fibre that promote the growth of beneficial bacteria [47–49]. Some dietary supplements, such as omega-3 fatty acids, are used in the treatment of depressive disorders [50], but most dietary supplements still lack scientific evidence [51]. Moreover, probiotic food supplements are now extensively tested as an add-on treatment for psychiatric disorders.

### Probiotics, Inflammation and the Vagus Nerve

The inflammatory hypothesis of psychiatric disorders has recently been the centre of attention; however, it is still uncertain where the chronic low-grade inflammation that characterises many psychiatric disorders actually originates [52]. SCFAs such as butyrate are important for gut barrier integrity and affect the CNS by altering the expression of brain-derived neurotrophic factor (BDNF). These SCFAs have been found to be of importance in psychiatric disorders; for example, they were found to be lower in depression [53]. SCFAs are vital for gut barrier function. A disruption in gut barrier integrity could lead to the translocation of bacteria and bacterial antigens (such as lipopolysaccharides) into the blood stream causing chronic low-grade inflammation [54].

To maintain homeostasis, the CNS responds constantly to environmental cues transmitted by the vagus nerve, which is one of the main players in MGBA communication. Peripheral cytokine production triggers the vagal anti-inflammatory reflex leading to production of acetylcholine which thereby prevents tissue damage by excessive cytokine release [55]. Recent research pointed out alterations of gut microbiota [56–60] as well as vagal tone in depressed individuals [61], patients with anxiety disorders [62] and schizophrenia [63]. Some probiotics, such as *Bifidobacterium* signal to the brain via vagal pathways [64, 65]. When the vagal nerve is cut, some probiotics no longer show effects on brain and behaviour [33, 66, 67].

### Probiotics to Modify the Gut-Brain Axis (Human Studies)

The gut microbiota impacts brain function, and an array of clinical studies provide us with insights into possible mechanisms. The clinical implications of probiotic use are currently under investigation for psychiatric indications. Recent trials of probiotic treatments yielded inconsistent results. Following a search with the relevant keywords, nine RCTs matched the inclusion criteria. Four RCTs included patients with schizophrenia, five RCTs included patients with depression and one RCT included patients with an anxiety disorder (generalised anxiety disorder). Table 2 gives an overview of RCTs published over the past 5 years (2014–2019), focusing on probiotics for the treatment of depression, anxiety and schizophrenia.

**Table 2:** Human randomised controlled trials (RCTs) published between 2014 and 2019 that investigated the effects of probiotics on symptoms of depression, anxiety and schizophrenia

Study reference	Region	Population/diagnosis/condition	Time of intervention	Intervention tested	Comparisons (sample size)	Outcomes
Schizophrenia						
Dickerson et al. [11]	USA	Schizophrenia or schizoaffective disorder (DSM-IV)	14 weeks	*Lactobacillus rhamnosus* strain GG (10^9^ CFU/day) and *Bifidobacterium animalis* subsp. *lactis* Bb12 (10^9^ CFU/day)	*N* = 58	PANSS
					F = 16	Bowel function no significant differences in the PANSS total score
					M = 42	
					Probiotic (*n* = 31)	
					Mean age, 44.4 years (11.0)	
					Placebo (*n* = 27)	
					Mean age, 48.1 years (9.4)	
Tomasik et al. [12]	USA	Schizophrenia or schizoaffective disorder (DSM-IV)	14 weeks	*Lactobacillus rhamnosus* strain GG (10^9^ CFU/day) and *Bifidobacterium animalis* subsp. *lactis* Bb12 (10^9^ CFU/day)	*N* = 58	PANSS
					F = 16	Systemic immunomodulatory effects of probiotic supplementation no significant differences in the PANSS total score
					M = 42	
					Probiotic (*n* = 31)	
					Mean age, 44.4 years (11.0)	
					Placebo (*n* = 27)	
					Mean age, 48.1 years (9.4)	
Severance et al. [13]	USA	Schizophrenia or schizoaffective disorder (DSM-IV)	14 weeks	*Lactobacillus rhamnosus* strain GG (10^9^ CFU/day) and *Bifidobacterium animalis* subsp. *lactis* Bb12 (10^9^ CFU/day)	*N* = 56	PANSS
					F = 19	Impact on yeast seropositivity (*Candida albicans* and *Saccharomyces cervisiae*) antibody levels and bowel discomfort; administration of probiotics may help normalise *Candida albicans* antibody levels and *Candida albicans*-associated gut discomfort in male individuals
					M = 37	
					Probiotic (*n* = 30)	
					Mean age, 44.66 years (11.4)	
					Placebo (*n* = 26)	
					Mean age, 48.11 years (9.6)	
						PANSS scores were not statistically altered in the longitudinal analyses
Ghaderi et al. [14]	Iran	Schizophrenia (DSM-IV) with disease duration of at least 2 years	12 weeks	50,000 IU of vitamin D3 and probiotic supplements containing *Lactobacillus acidophilus*, *Bifidobacterium bifidum*, *Lactobacillus reuteri* and *Lactobacillus fermentum* (8 × 10^9^ CFU/day)	*N* = 60	PANSS
					F = 4	Biomarkers of oxidative stress and inflammation, lipid profiles and glycaemic control
					M = 56	
					Probiotic + vitamin	
					D (*n* = 30)	
					Mean age, 44.8 years (8.3)	
					Placebo (*n* = 30)	
					Mean age, 43.2 years (6.0)	
						significant effect of probiotics and Vitamin D on total PANSS Score (p = 0.007) but no impact on negative and positive PANSS sub-scores
Depression						
Akkasheh et al. [15]	Iran	Major depression (DSM-IV)	8 weeks	*Lactobacillus acidophilus* (2 × 10^9^ CFU/g), *Lactobacillus casei* (2 × 10(9) CFU/g) and *Bifidobacterium bifidum* (2 × 10^9^ CFU/g)	*N* = 40	BDI and metabolic parameters (fasting plasma glucose, insulin metabolism, lipid concentrations, hs-CRP, oxidative stress)
					F = 34	
					M = 6	
					Probiotic (*n* = 20)	
					Mean age, 38.3 years (12.1)	
						Probiotic administration had beneficial effects on BDI, insulin, homeostasis model assessment of insulin resistance, hs-CRP concentrations and glutathione concentrations
					Placebo (*n* = 20)	
					Mean age, 36.2 years (8.2)	
Kazemi et al. [16]	Iran	Major depression (mild to moderate depression, diagnosed by a psychiatrist)	8 weeks	*Lactobacillus helveticus* R0052 (2 × 10^9^ CFU/g) and *Bifidobacterium longum* R0175 (2 × 10^9^ CFU/g), prebiotic (galactooligosaccharide)	*N* = 110	BDI
					F = 78	Serum tryptophan and BCAAs, kynurenine improvement in BDI score compared with placebo whereas no significant effect of prebiotic supplementation was seen; kynurenine/tryptophan ratio decreased significantly in the probiotic group compared with the placebo group after adjusting for serum isoleucine (*p* = 0.048). tryptophan/isoleucine ratio increased significantly in the probiotic group when compared with placebo (*p* = 0.023).
					M = 32	
					Probiotic (*n* = 36)	
					Mean age: 36.15 years (7.85)	
					Placebo (*n* = 38)	
					Mean age, 36 years (8.47)	
					Prebiotic (*n* = 36)	
					Mean age, 37.35 years (7.97)	
Majeed et al. [17]	India	Major depression (DSM-IV) and Rome III Diagnostic Criteria for Functional IBS	90 days	*Bacillus coagulans* MTCC 5856 (2 × 10^9^ CFU)	*N* = 40	HAMD, MADRS, CES-D and IBS-QOL
					F = 34	
					M = 6	Significant improvement of HAMD (*p* = 0.005), MADRS (*p* = 0.007), CES-D (*p* = 0.009) and IBS-QOL (*p* = 0.010) in the intervention group with *Bacillus coagulans* MTCC after 90 days in comparison with the placebo group. There were no significant differences of HAMD, MADRS, CES-D and IBS-QOL in the placebo group after 90 days.
					Probiotic (*n* = 20)	
					Mean age, 40.36 years (10.28)	
					Placebo (*n* = 20)	
					Mean age, 43.88 years (9.85)	
						Serum myeloperoxidase, an inflammatory biomarker was significantly reduced (*p* < 0.01) in the probiotic group in comparison with the placebo group after 90 days.
Pinto-Sanchez et al. [18]	Canada	Mild to moderate anxiety and/or depression (HAD-A or HAD-D score 8–14) and IBS with diarrhoea or mixed-stool pattern (Rome III criteria)	6 weeks	*Bifidobacterium longum* NCC3001 (1.0E+10 CFU/1 g powder with maltodextrin)	*N* = 44	Hospital Anxiety and HADS-A and HADS-D
					F = 24	
					M = 20	Depression scores were reduced compared with placebo.
					Probiotic (*n* = 22)	
					Mean age, 46.5 years (30–58) IQR	
					Placebo (*n* = 22)	
					Mean age, 40.0 years (26–57) IQR	
Chahwan et al. [19]	Australia	Mild to severe depression (BDI > 12)	8 weeks	*Bifidobacterium bifidum* W23, *Bifidobacterium lactis* W51, *Bifidobacterium lactis* W52, *L. acidophilus* W37, *Lactobacillus brevis* W63, *Lactobacillus casei* W56, *Lactobacillus salivarius* W24, *Lactococcus lactis* W19, *Lactococcus lactis* W58 (total cell count 1 × 10^10^ CFU/day)	*N* = 71	BDI
						DASS
					F = 49	BAI
						No significant differences in BDI, DASS and BAI.
					M = 22	
					Probiotic (*n* = 34)	
					Mean age, 36.65 years (11.75)	
					Placebo (*n* = 37)	
					Mean age, 35.49 years (12.34)	
Anxiety						
Eskandarzadeh et al. [20]	Iran	Generalised anxiety disorder (DSM-V criteria)	8 weeks	18 × 10^9^ CFU *Bifidobacterium longum*, *Bifidobacterium bifidum*, *Bifidobacterium lactis* and *Lactobacillus acidophilus*	*N* = 48	HAM-A
					F = 39	STAI
						BAI
						HAM-A decreased more in the probiotics + sertraline (PS) group (*p* = 0.003). Although the reduction of BAI was also more in the PS group, it was not significantly different from that of the sertraline alone (S) group. Moreover, despite the greater reduction of State-Anxiety Inventory score in the PS group, the score of Trait-Anxiety Inventory was not statistically different between the 2 groups at week 8.
					M = 9	
					Sertraline + probiotic (*n* = 24)	
					Mean age, 34.17 years (6.14)	
					Sertraline + placebo (*n* = 24)	
					Mean age, 33.67 years (6.56)	

*F*, number of female participants; *M*, number of male participants; *DSM*, *Diagnostic and Statistical Manual of Mental Disorders*; *CFU*, colony forming unit; *PANSS*, Positive and Negative Symptom Scale; *BDI*, Beck Depression Inventory; *hs-CRP*, high sensitive C-reactive protein; *BCAA*, branched chain amino acid; *HAMD*, Hamilton Rating Scale for Depression; *MADRS*, Montgomery-Asberg Depression Rating Scale; *CES-D*, Center for Epidemiological Studies Depression Scale; *IBS-QOL*, irritable bowel syndrome quality of life questionnaire; *HADS-A*, Hospital Anxiety and Depression Scale-Anxiety; *HADS-D*, Hospital Anxiety and Depression Scale-Depression; *HAM-A*, Hamilton Rating Scale for Anxiety; *BAI*, Beck Anxiety Inventory; *STAI*, StateTrait Anxiety Inventory; *DASS*, Depression Anxiety Stress Scale; *IQR*, interquartile range

### Probiotics and Depression

Major depression is among the most prevalent disorders worldwide and therefore is of utmost importance in the context of health policy [68]. Patients with depression show significant differences in gut microbiota composition in comparison with those without depression [56–60, 69]. When rats are colonised with faecal matter from patients with depression, they exhibit depressive-like symptoms [58]. However, there is no specific ‘dysbiosis’ signature found in depression. A variety of studies have investigated probiotic effects on mood. Most of them have been done in healthy populations or in participants without an adequately diagnosed depressive disorder. To date, several meta-analyses support the use of probiotics to improve mood [70–73]. However, mood effects are only significant in participants exhibiting symptoms of depression [72].

Currently, there are five probiotic RCTs using predominantly *Lactobacillus* and *Bifidobacterium* species to treat depression (see Table 1). Akkaseh et al. included 40 participants with major depressive disorder (MDD) in the probiotic RCT [15]. After 8 weeks, the 20 patients in the active intervention group showed significantly decreased BDI scores in comparison with the placebo group.

Another RCT from Kazemi et al. included 110 participants, with 36 receiving a probiotic, 38 receiving placebo and 35 receiving a prebiotic [16]. After 8 weeks of supplementation, the probiotic group showed a significant reduction of the BDI score in comparison with the placebo and probiotic supplementation group.

Majeed et al. included 40 patients with a co-diagnosis of MDD and irritable bowel syndrome (IBS). Twenty were allocated to the probiotic group and twenty to the placebo group for a 90-day intervention. After the intervention, the probiotic group showed a significant improvement on the depression scales (Hamilton Rating Scale for Depression (HAMD), Montgomery–Åsberg Depression Rating Scale (MADRS), Center for Epidemiologic Studies Depression Scale (CES-D)). However, in this study, clear conclusions regarding patients with MDD cannot be made, because of the co-diagnosis of IBS.

A significant reduction of depression scores but not anxiety scores was found in the RCT of Pinto-Sanchez et al. [18], after a 6-week treatment of 22 patients receiving *Bifidobacterium longum* in comparison with 22 patients receiving placebo.

The latest study of Chahwan did not find a significant effect on depressive symptoms following an 8 week intervention with a multi-strain probiotic [19].

However, all these studies lacked gut microbiota profiling of patients before and after probiotic use. Moreover, these studies show some discrepancies regarding strains and duration of treatment (reaching from 6 to 13 weeks). Three of five studies used combinations of *Lactobacillus* and *Bifidobacterium* species [15, 16, 19], while two of five studies used single strains such as *Bifidobacterium longum* [18] and *Bacillus coagulans* [17]. Due to the paucity of studies, direct conclusions on the optimal strain combinations and duration of treatment cannot be drawn. However, long-term probiotic supplementation may have some merit as probiotics cannot be detected in stool 1–4 weeks after the consumption is stopped [74]. For example, in the study of Pinto-Sanchez, depression scores were still significantly better compared with baseline in the follow-up (4 weeks after the end of the probiotic intervention), but the depression scores were rising again [18].

### Probiotics and Anxiety

There have been multiple studies examining the effects of probiotics on anxiety symptoms in other diseases such as IBS (for a review and meta-analysis, see, [75]). In animal studies, stress, HPA axis response and anxiety-related behaviour were affected after probiotic intake [76, 77]; however, results were often inconsistent [75].

To our best knowledge there is only one single publication reporting RCT data of a probiotic to treat patients with a diagnosed anxiety disorder (generalised anxiety disorder (DSM-V criteria)) [20•]. This small Iranian RCT tested the impact of an 8-week intervention with a multi-strain probiotic containing *Bifidobacterium longum, Bifidobacterium bifidum*, *Bifidobacterium lactis* and *Lactobacillus acidophilus*. Twenty-four patients were assigned to the probiotic intervention group and twenty-four to the control group. Probiotic and placebo capsules were given as an add-on therapy as both the control and probiotic intervention group received a baseline selective serotonin reuptake inhibitor (SSRI) therapy with sertraline. They used the Hamilton Rating Scale for Anxiety (HAM-A), the Beck Anxiety Inventory (BAI) and the State Trait Anxiety Inventory (STAI) to quantify anxiety symptoms before and after the probiotic intervention. After 8 weeks, there was a significant reduction in the HAM-A score in the group receiving probiotics and sertraline in comparison with the sertraline plus placebo group. However, the BAI score was not significantly different. After 8 weeks, only state anxiety was different in the group receiving sertraline plus probiotic but not trait anxiety. In relation to biological markers, the researchers measured ACTH and serum cortisol levels. These parameters did not significantly change in either the intervention or the control group.

Unfortunately, there have been no other interventional studies in people with clinically relevant anxiety disorders. Further research in this area should be done given this small but encouraging trial and the ever-expanding literature outlining promising preclinical results.

### Probiotics and Schizophrenia

Schizophrenia is mainly a heritable disorder; however, many researchers assume a possible aetiological role of the gut microbiome through epigenetic modulation (i.e. diet and exposure to infectious agents), influence on the immune system and neuroinflammation [78, 79]. Interestingly, many of the genetic loci related to schizophrenia are known to modulate the immune system and inflammation [80]. Moreover, central neurotransmitter changes were found in mice after receiving a FMT from patients with schizophrenia [81].

For schizophrenia, there are three RCTs, which were already systematically reviewed by Ng et al. They did not find a significant difference in schizophrenia symptoms between probiotic and placebo groups postintervention when applying a per-protocol analysis and a fixed effects model [82].

All of these three studies were from the same research group. They included patients with schizophrenia or schizoaffective disorder and tested the same intervention (multistrain-probiotic containing *Lactobacillus rhamnosus* and *Bifidobacterium animalis* subsp*. lactis*). With this probiotic no significant effects on the Positive and Negative Symptom Scale (PANSS) total score (*p* = 0.25) could be found after 14 weeks of intervention in all three studies. However, Dickerson et al. reported a reduced risk for severe bowel problems in patients with moderate to severe schizophrenia symptoms after treatment with the probiotic supplement (*p* = 0.003) [11]. Tomasik et al. found systemic immunomodulatory effects (via cytokine modulation) of probiotic supplementation (reduction of the acute-phase reactant von Willebrand factor, *p* = 0.047). Another RCT by Severance et al. showed an inverse link of *C. albicans* antibody level with GI symptoms in patients with schizophrenia. The most recent study tested a probiotic supplement in combination with vitamin D3 [14]. Ghaderi et al. showed a significant effect of a 12-week intervention on the PANSS score (*p* = 0.007); however, there was no impact of the intervention on PANSS subscores.

## Conclusions—Probiotics as Modifiers of the Gut-Brain Axis

In this review, we summarise important clinical findings regarding the MGBA and results from recent RCTs focusing on probiotic interventions for psychiatric disorders. Probiotics appear to have an impact on symptoms of depression but not schizophrenia. As there is only one RCT so far using a probiotic as an adjunctive treatment for anxiety, no firm conclusions can be drawn.

The MGBA provides the field of psychiatry a new paradigm for the treatment of mental illness. Despite receiving up-to-date, evidence-based, multimodal treatments, many psychiatric patients continue to experience distressing symptoms. Even with conflicting clinical results, probiotic use is greatly popularised in the media and probiotics belong to the most commonly consumed food supplements [83].

It should be mentioned that, although modulation of the MGBA with probiotics appear promising as a therapeutic strategy for mental illness, several challenges remain. First, RCTs published to date display comparably small sample sizes and methodological heterogeneity. Many studies also only use self-reported parameters of symptomatology without a sufficient assessment of subjects or to confirm a clinical diagnosis and screen for comorbidities. Secondly, probiotics may not work in the same way for every patient. For example, a recent study from Washington State University has shown that under certain conditions, ingested probiotics could evolve and adapt in either a positive or a negative way according to the given environment in the gut. As living organisms, probiotics are subjected to natural selection. For example, the probiotic bacterium *Escherichia coli* Nissle was found to enhance mucin utilisation in low-diversity environments which could damage the intestinal lining [84].

Another important contributor to the high variability of results in probiotic studies is the variety of studied strains and strain combinations. For example, different strains of the same species have demonstrated opposing effects in relation to psychological symptoms: while *Lactobacillus rhamnosus* (strain JB-1) did not affect mood or anxiety levels in healthy men [85], *Lactobacillus casei* (strain Shirota) improved mood in participants with low baseline mood scores [86].

This underlines the necessity of combining probiotics with a diet containing an adequate amount of micro- and macronutrients to promote favourable development of the gut flora. Notably, individuals suffering from psychiatric illness, and especially individuals with schizophrenia, show poor dietary patterns [87]. Furthermore, the gut microbiome can also be altered by certain psychotropic medications, which should be taken into account [88•]. In particular, antidepressants and antipsychotics could alter the gut microbiota of the host [89–91].

A species and strain-sensitive assessment of participants evaluated mucosal colonisation after consumption of 11 probiotic strains and found that 40% of the tested individuals showed a near-total colonisation resistance after probiotic ingestion and the degree of mucosal association could be predicted by baseline host and microbiome factors [92]. In light of this, an unresolved issue is whether gut colonisation by probiotics is stable or merely a transient event [92]. Further research should focus on individual, personalised approaches including a targeted therapy with pre- and probiotics according to the gut environment of the individual. This therapy should also take environmental factors (diet, fluid intake, age, gender, comorbidities) into account.

Against this background, the area of nutrition and gut health will likely become an important component in the biopsychosocial treatment model in psychiatry. The evolving field of nutritional psychiatry should therefore be integrated in clinical practice to treat and prevent psychiatric disorders as well as metabolic comorbidities [93].

In summary, probiotics could be used as an add-on treatment for some psychiatric indications such as depression; however, as effect sizes are low, they are unlikely to substitute psychopharmacological approaches in the future. Especially for anxiety disorders, the evidence is very weak, and there is still a huge research gap which needs to be filled in the years to come.
